# Monitoring of contamination by microplastics on sandy beaches at Vulcano Island (Sicily, Italy) by hyperspectral imaging

**DOI:** 10.1007/s11356-024-34972-6

**Published:** 2024-09-25

**Authors:** Pietro Cocozza, Silvia Serranti, Andrea Setini, Paola Cucuzza, Giuseppe Bonifazi

**Affiliations:** 1 Department of Chemical Engineering, Materials and Environment, Sapienza University of Rome, Via Eudossiana 18, 00184 Rome, Italy; 2 Department of Biology and Biotechnology “Charles Darwin, Sapienza University of Rome, Section of Zoology Viale Dell’Università, 32, 00185 Rome, Italy

**Keywords:** Plastic litter, Aeolian Islands, Polymer analysis, Environmental pollution, Microplastics Diversity Integrated Index

## Abstract

In this work, the monitoring and characterization of large microplastics (1–5 mm) collected from sandy beaches of Vulcano Island (Aeolian Islands, Sicily, Italy) were carried out for the first time. Microplastics were sampled from two beaches, “Gelso” and “Sabbie Nere,” in three different time periods. The following characteristics of microplastic samples were assessed: quantity, distribution, categories, color, polymer type, size, and shape parameters. The polymers were identified using hyperspectral imaging, whereas an automatic image analysis approach was employed to determine microplastics’ morphological and morphometrical attributes. Finally, the microplastic diversity integrated index was computed to obtain information on the potential emission sources of microplastics. It was found that the concentration of microplastics varies from 0.27 particles/kg_dw to 1.35 particles/kg_dw with fragment being the main collected category, with minor amount of pellet, foam, film, and filament. The predominant color of microplastics was by far white, followed by blue and yellow. The identified polymers were polyethylene and polypropylene followed by expanded polystyrene, polyamide, polystyrene, and polyethylene terephthalate. The morphological and morphometrical characterization highlighted a large variability for most size and shape parameters. Finally, the Microplastics Diversity Integrated Index results showed average indices compared to the literature, with higher values for the “Gelso” site (0.656), indicating a higher heterogeneity of sources, with respect to “Sabbie Nere” beach (0.530).

## Introduction

The growing global issue of plastic pollution increasingly involves marine and coastal environments (Iannilli et al. 2018; Mistri et al. 2021; Uy and Johnson 2022; Corti et al. 2023; Ditlhakanyane et al. 2023; Osman et al. 2023; Tiwari et al. 2023). In 2022, global plastic production reached 400.3 Mt (Plastics Europe 2023), and the recent COVID-19 pandemic contributed to its increase, aggravating the issue of long-lasting marine debris. If these debris are caught by ocean currents, they become part of vast oceanic waste patches, commonly known as plastic islands. Plastic, in this way, ends up in the fish we consume (Ditlhakanyane et al. 2023) and, through the water cycle, in the water we drink (Chowdhury et al. 2023).

To address this issue, halting the flow of plastic into the oceans by reducing sources of input is crucial, also in agreement with the Sustainable Development Goals (SDGs) of the 2030 Agenda, in particular with SDG14 “Life Below Water,” followed by SDG3 “Good Health and Well-being,” and SDG6 “Clean Water.”

The sea has a fundamental role in human life on Earth. Besides threats such as overfishing, climate change, ocean acidification, and habitat destruction, marine ecosystems are also affected by pollution (Dubinsky and Stambler 1996; Andrady 2017; Abou Samra and Ali 2024; Pásková et al. 2024; Yu et al. 2024). For these reasons, the biodiversity in the oceans is decreasing, disrupting food chains of both marine and terrestrial environments (Damak et al. 2019). Among the various types of marine pollution, one of the most problematic for ecosystems is that caused by microplastics (MPs).

MPs are polymeric items characterized by an average diameter of less than 5 mm (Barnes et al. 2009; Andrady 2017). This size range is very wide and includes particles characterized by great variability in terms of morphometric and morphological parameters. For this reason, a comparative evaluation of microplastic concentrations, starting from literature data, is quite difficult. As suggested by the European Commission (2013), a categorization was introduced to classify small microplastics (SMPs: <1 mm) and large microplastics (LMPs: 1–5 mm) (Van Cauwenberghe et al. 2015).

MPs are considered of primary origin (intentionally produced, entering the environment already with a dimension of MPs) or secondary origin (resulting from the degradation of bigger plastic items) (Andrady 2017). In addition, MPs are usually subdivided into several categories based on their shape: fragments, pellets, fibers, films, foam, and granules (Löder and Gerdts 2015; Anderson et al. 2017).

The most common methods for polymer classification are Fourier-transform infrared (FT-IR) spectroscopy and Raman spectroscopy (Klein et al. 2018; Tirkey and Upadhyay 2021). Such techniques are based on detecting the various absorption energies of polymer functional groups and are capable of measuring both at a specific point and through image mapping, requiring significant analysis time (Käppler et al. 2016; Xu et al. 2019).

The long analysis times of the aforementioned instruments are leading to the exploration of new polymer analysis techniques. In the last years, hyperspectral imaging (HSI) started to be applied to perform MPs identification and characterization (Serranti et al. 2018, 2019; Huang et al. 2021; Fiore et al. 2022). HSI is a non-invasive and non-destructive technique combining advantages of spectroscopy with those of image analysis. In fact, a spectrum for each pixel of the image can be acquired in a fast and effective way, obtaining a cube of data (i.e., hypercube), having two spatial dimensions (the image size) and one spectral dimension (the wavelength). Both wavelength intervals and spatial resolution can be set up according to the application.

The wavelength interval in the near- (NIR: 1000–1700 nm) and short-wave infrared (SWIR: 1000–2500 nm) ranges provides useful information for the recognition of the different polymers, according to the absorption features (combination and overtone bands) (Serranti et al. 2020; Bonifazi et al. 2022, 2023). HSI allows to strongly reduce the time requested for the analysis, compared to the more widely used technologies, thanks to the possibility of rapid investigation of large areas, without preliminary sample preparation. Furthermore, the device cost is much lower than that of the common analytical techniques used for microplastic identification.

After the characterization of polymer, category, and color of microplastic particles, it is possible to calculate the Microplastic Diversity Integrated Index (MDII) proposed by Li et al. (2021). MDII was introduced to highlight MP differences, to define the complexity of pollution sources, and to compare successive data from the same site or data from different sites. MDII is also influenced by the distance and intensity of the emission source. Hence, when MDII in a site registers a high value, it suggests the presence of multiple sources of microplastic pollution, providing more or less the same contribution. Conversely, a low value of MDII indicates either a scarcity of pollution sources or a multitude of sources with variable contributions (Li et al. 2021).

MPs represent a threat to aquatic organisms because, if ingested, they can release toxic substances adsorbed on the surface or present inside the polymer, causing double damage. MPs are diffused in the different environments, such as atmosphere (Mbachu et al. 2020; Dehhaghi and Pardakhti 2023), agricultural soils (Koyuncuoğlu and Erden 2021; Henseler et al. 2022), freshwater (Sighicelli et al. 2018; Fiore et al. 2022; Chowdhury et al. 2023), and especially in the marine environment at every level: surface seawater (Kanhai et al. 2020; Xie et al. 2021), water column (Cincinelli et al. 2019), deep water (Pinheiro et al. 2023; Yücel 2023), aquatic organisms (Uy and Johnson 2022; Ditlhakanyane et al. 2023), terrestrial organisms living on coasts (Iannilli et al. 2018; Osman et al. 2023), seafloor sediment (Mistri et al. 2021; Corti et al. 2023), and coastal sandy sediment (Tiwari et al. 2023).

Moreover, MPs are currently present in the human food chain (Smith et al. 2018; Senathirajah et al. 2021; Battistin et al. 2023) with a high risk to human health (Blackburn and Green 2022; Osman et al. 2023; Tuna et al. 2023).

The negative effects of MPs are particularly intensive in semi-enclosed basins with strong anthropization, such as the Mediterranean Sea (Cózar et al. 2015). The latter is considered one of the main biodiversity reservoirs, being characterized by the presence of 7.5% and 18% of the world’s marine fauna and flora, respectively, in just 0.7% of the ocean (Hassen et al. 2023). The richness in biodiversity of the Mediterranean Sea is due to its fundamental role in the migration and reproduction of many animal species, facilitated by the presence of various islands offering abundant nutritional resources. One of the most important biodiversity spots is provided in the Tyrrhenian Sea by the Aeolian Islands. Different studies have been carried out at the level of the Tyrrhenian Sea to investigate macro, meso, and microplastics both quantitatively and qualitatively. However, no studies have been carried out on MPs in the coastal sandy sediments of Vulcano Island (Aeolian Islands, Italy) at multiple sampling points.

The main aims of this work are (i) to conduct pioneering monitoring efforts aimed at detecting the presence of LMPs in the sediment of Vulcano Island and (ii) to analyze the origin of the sampled LMPs, thus providing insights into their sources and distribution patterns, based on the characterization of the collected LMP samples in terms of concentration, category, color, polymer, and morphological and morphometrical parameters.

## Materials and methods

### The investigated sites

**Fig. 1:**
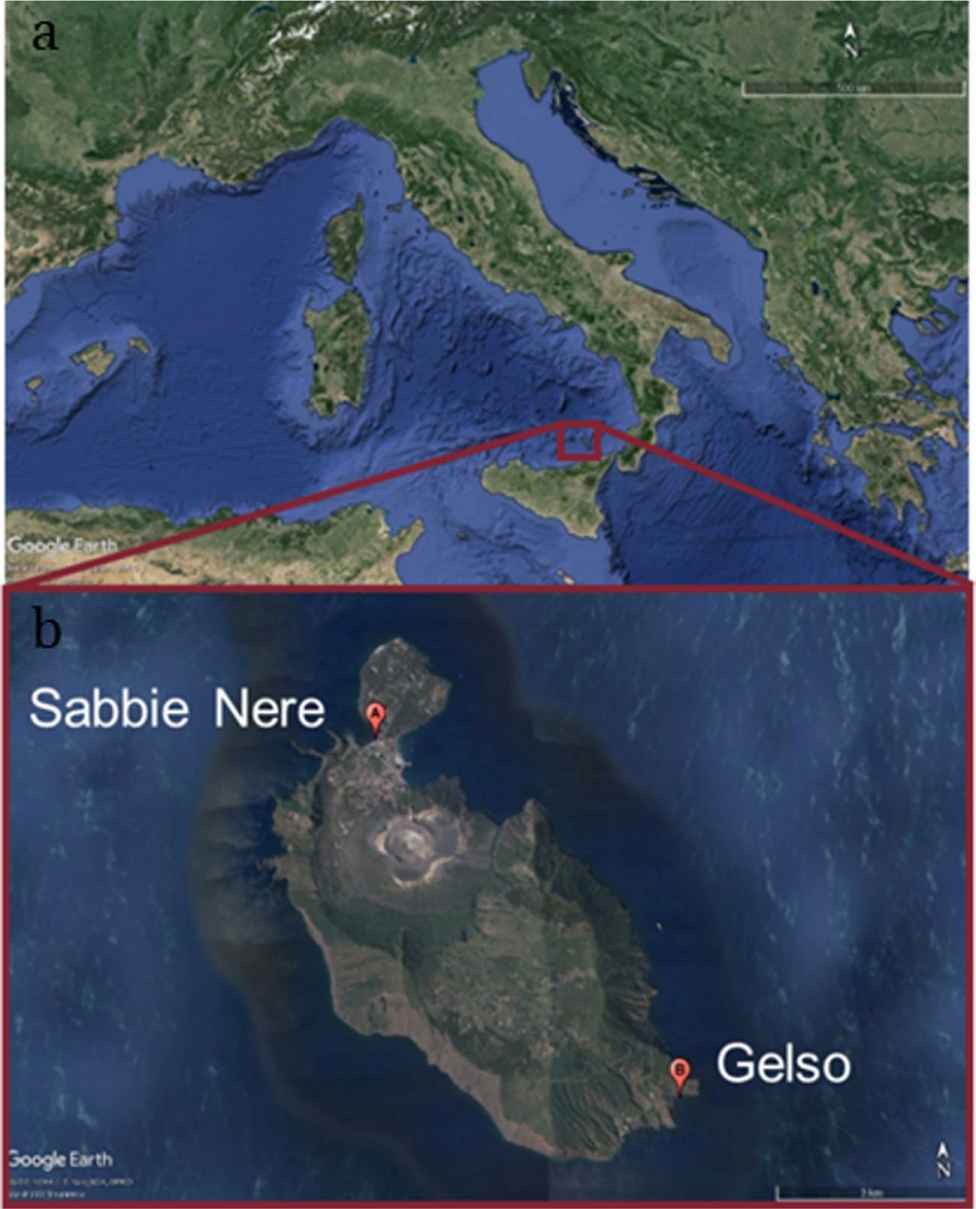
**a**) General overview of Vulcano Island on Italy map; **b**) sampling sites on the island of Vulcano

The island of Vulcano is part of the Aeolian Islands archipelago, along with Lipari, Salina, Panarea, Stromboli, Alicudi, and Filicudi (Fig. 1).

The Aeolian Archipelago is in the southern Tyrrhenian Sea, between 38°21′54″ and 38°48′40″ North latitude, 14°20′35″ and 15°14′70″ East longitude. The Aeolian Archipelago, an esteemed UNESCO World Heritage Site, has been selected for the establishment of a marine protected area, further fortifying its conservation and safeguarding its rich biodiversity and cultural significance (MPA) (L.979/82).

Geographically, the island of Vulcano is the closest island to Sicily. From a geological perspective, it has a volcanic origin, composed of effusive rocks (Di Traglia et al. 2013). Vulcano is home to about 600 residents; the majority live in the part called Vulcano Piano, situated at approximately 400 m above sea level. There are three sandy beaches on the island easily accessible from the land. Two beaches, Sabbie Nere and Acque Calde, are very close to the urbanized area and are part of an isthmus that connects the Vulcanello locality in the north with the rest of the island. The third sandy beach, Gelso, is located near the small fishing village at the southeastern tip of the island, the farthest from inhabited centers. In this work, Sabbie Nere and Gelso beaches (Fig. 1) were investigated, being located on opposite sides of the Island and having different anthropic pressures.

#### Sabbie Nere

It is a beach that is 500 m long and 20–30 m wide. Typically, the dimensions of the beach remain constant throughout the year. The exposure is to the northwest. The first area of land encountered in the northwest direction after this bay is Sardinia (500 km away).

Tourism is highly prevalent between the months of May and October, with the peak occurring in August.

#### Gelso

It is a small beach, about 150 m long and 25 m wide, facing south, with Sicily about 10 miles away. Summer tourism on this beach is much less than on the Sabbie Nere site.

The size of the beach is not constant throughout the year. Sand deposition and erosion vary so significantly that in some years the beach is almost non-existent, while in others, it can extend up to 50 m wide.

### Microplastic sampling

Microplastic sampling was carried out in three different periods during 1 year, every 4 months: July 2021, November 2021, and March 2022. These periods were chosen because they coincide with the beginning of the tourist season (March), the peak period of tourist activity (July), and the end of the tourist season (November).

**Fig. 2:**
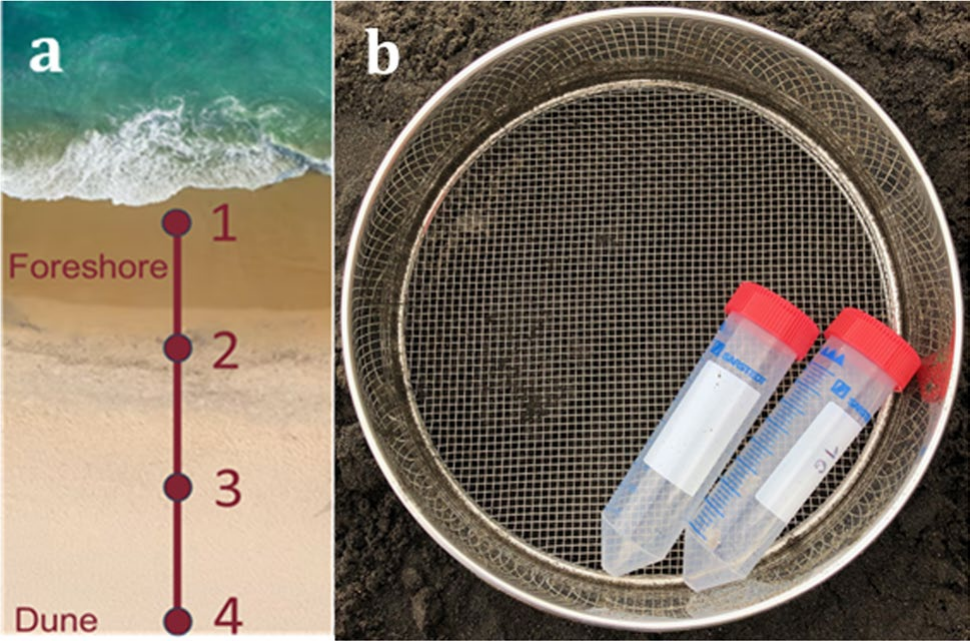
**a**) Transect perpendicular to the coastline; **b**) sieve with a 1 mm mesh

Each sampling was carried out along a transect perpendicular to the shoreline, consisting of 4 points spaced 5 m apart from each other, extending from the foreshore to the dune (Fig. 2a).

For each point, a portion of sand measuring 0.025 m^3^ (50 × 50 × 10 cm in depth) was initially sieved with a 5-mm mesh sieve and then with a 1-mm mesh sieve (Fig. 2b). This allowed the collection of large microplastics (LMPs), ranging in size from 1 to 5 mm. During sieving, a careful analysis was carried out to remove biological matter. The LMPs were collected using tweezers and subsequently analyzed in the laboratory. This procedure was recommended for the sampling of LMPs by the European Commission (2013).

The concentrations have been expressed as the number of plastic particles per dry weight of sediment (particles/kg_dw), following the suggestion of Razeghi et al. (2021).

During the sampling process, it was crucial to minimize contamination by avoiding contact with plastic materials, including during sample transportation. All samples, from the collection to the arrival/delivery at the laboratory, were stored and transported under stable conditions and reached the laboratory within a maximum of 48 h. The containers used for sampling are made of glass.

### Laboratory analysis of microplastics

Microplastic samples were analyzed at the Raw Materials Laboratory (RawMaLab) of the Department of Chemical Engineering, Materials and Environment (DICMA), Sapienza University of Rome.

**Fig. 3:**
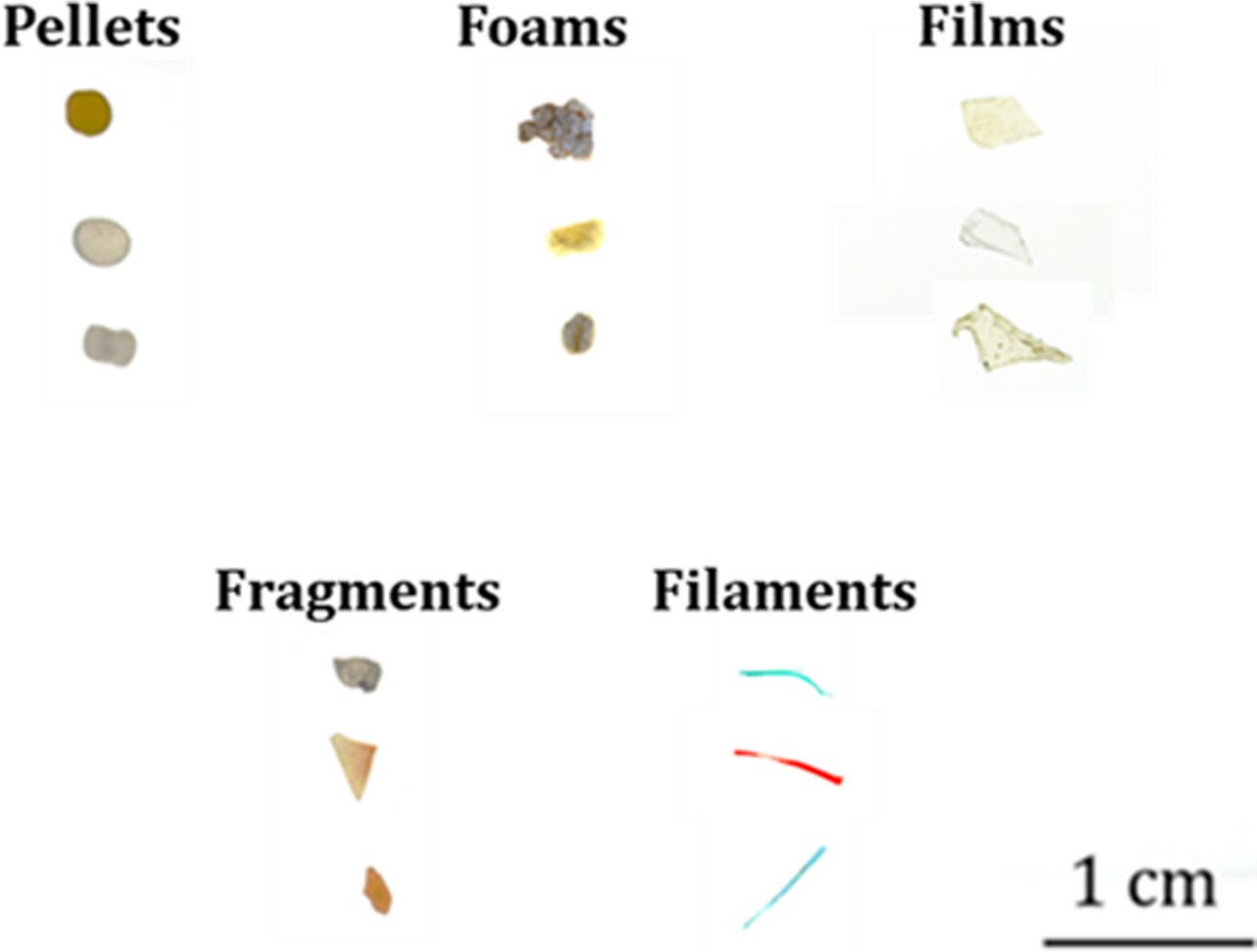
Example of digital pictorial image of LMPs acquired by Nikon D5200 camera

The LMPs were counted and classified based on category, color, polymer, and morphological and morphometrical parameters. The microplastic category and color were visually defined by an operator. The polymers of microplastic samples were classified using HSI. Additionally, for each investigated sample, a digital pictorial image was acquired (Fig. 3) through a Nikon D5200 camera for documentation purposes and subsequent morphological and morphometric analysis.

To avoid contamination, MP samples were carefully handled, in accordance with established guidelines (Cowger et al. 2020); during LMP selection, unused samples were adequately covered with glass; only glass and metal objects were utilized, excluding plastic materials or devices. Furthermore, only the technician performing the work was inside the laboratory, wearing 100% cotton clothes.

#### Polymer identification: hyperspectral imaging analysis

To perform polymer recognition, LMP samples were acquired using the hyperspectral platform SISUChema XL^TM^ Chemical Imaging Workstation embedding the ImSpector^TM^ N25E spectrometer (Specim Ltd., Oulu, Finland), working in the SWIR range (1000–2500 nm). The LMP particles were arranged on a small conveyor belt and captured line by line through the “31-mm” lens. A field of view of 50 mm was acquired, characterized by a spatial and spectral resolution equal to 156 µm/pixel and 6.3 nm, respectively. Acquisition speed was set at 17.35 mm.

This technology allows the collection of spectral information from a material, for each pixel of the acquired image, in a non-destructive manner through the interaction between light and the investigated object. The results of this interaction can be detected through the spectral signature of the material, characterized by specific absorptions in different regions of the investigated spectrum. These absorptions are related to the molecules of the sample and, specifically, to their vibrational motions.

The acquired SWIR-HSI data were handled using MATLAB® environment (Version 9.3.0, The Mathworks, Inc.) and then processed by the PLS_toolbox (version 8.6; Eigenvector Research, Inc.).

Collected data were preprocessed in order to remove the image background; the Mean Center algorithm (Rinnan et al. 2009) was utilized to reach this goal. Principal Component Analysis (PCA) was subsequently applied for exploratory purposes, aiming to evaluate the potential recognition of the different polymers. Finally, a hierarchical PLS-DA (Partial Least Squares-Discriminant Analysis) classification model, as previously described in Fiore et al. (2022), was employed. In particular, the applied classification model enables the identification of the following polymers: expanded polystyrene (EPS), polyamide (PA), polyethylene (PE), polyethylene terephthalate (PET), polypropylene (PP), and polystyrene (PS).

#### Morphological and morphometrical characterization

Digital images of MP samples were acquired using a Nikon D5200 camera. These images were then processed using the software MATLAB® R2022a (version 9.12, The Mathworks, Inc.) to obtain the morphological and morphometrical characterization.

The procedure for morphological and morphometric characterization, as outlined by Fiore et al. (2022), comprises four steps: (1) spatial calibration, (2) image binarization, (3) particle counting and labeling, and (4) measurements of the morphological and morphometric parameters.

MPs were characterized through the evaluation of the following parameters: Area of the particle (mm^2^); Aspect (ratio between major and minor axes of the ellipse equivalent to the particle), providing information on the elongation of particles; Perimeter of the particle (mm); Minimum, Mean, and Maximum Feret Diameter (mm); Roundness, giving information related to the particle circularity (a circle has the value of roundness = 1) and computed using the following equation:

$$\frac{{\left(\text{Perimeter}\right)}^{2}}{4\pi Area}$$

### MDII

To obtain the MDII, Simpson’s Diversity Index (Simpson 1949) was calculated for the shape, color, and polymer of all collected LMPs. As mentioned earlier, this index was proposed to evaluate the composition of the MP population and to provide information on the pollution source.

Based on the above, the calculation formula for the MDII developed by Li et al. (2021) is as follows:

$${\text{MDII}=(\text{Simpson}\_\text{shape}\times \text{Simpson}\_\text{color}\times \text{Simpson}\_\text{polymer})}^{1/3}$$

The index ranges from 0 to 1, where a value closer to 0 indicates lower MP diversity, while a value closer to 1 suggests higher MP diversity (Li et al. 2021).

## Results and discussion

### Microplastic abundances, categories, and colors

**Fig. 4:**
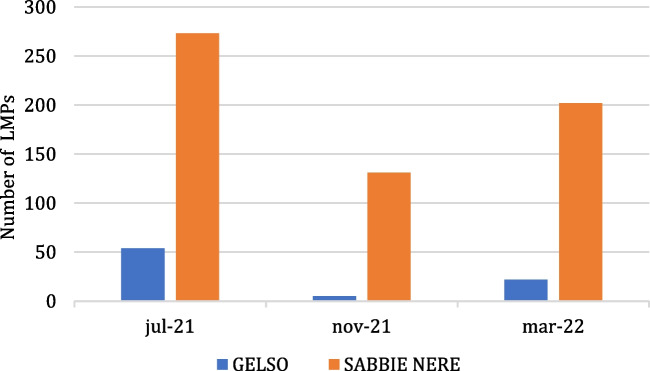
Quantity of LMPs in the three sampling periods for the two investigated beaches

The total collected LMPs amount to 687 particles; the majority of which were found at Sabbie Nere (606 particles), with only 81 particles at Gelso. The seasonal distribution of LMPs, shown in Fig. 4, reveals a consistent pattern between Sabbie Nere and Gelso. In fact, in July 2021, the number of particles is the highest at each site, followed by March 2022 and lastly by November 2021.

The average abundance of sampled LMPs on the island of Vulcano amounts to 0.92 particles/kg of dry sediment. The highest value was registered at Sabbie Nere with 1.35 particles/kg of dry sediment, while the lowest was at Gelso with 0.27 particles/kg of dry sediment. Certainly, the fact that it is an island and there are no major shipping routes nearby may contribute to keeping the area uncontaminated; however, the high tourism could be the cause of the presence of plastics found on the territory of the island of Vulcano. As evidence of this, at Sabbie Nere, the beach most frequented by tourists and the one closest to the town center, the presence of LMPs is consistently higher than at Gelso. Correlation between tourism and the presence of MPs is also supported by Gül (2023).

**Fig. 5:**
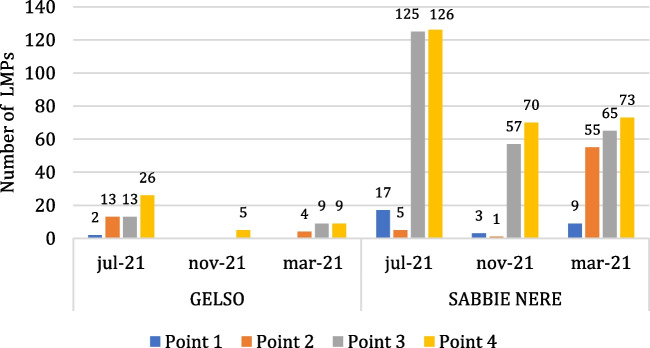
Distribution of LMPs along the two beaches’ profile

In addition to the difference found between sampling sites, it is also important to consider the variation among transect sampling points, as shown in Fig. 5.

At both sites, a similar distribution occurs: from the foreshore towards the dune, there is an increasing concentration of LMPs. The point with the highest number of LMPs is Point 4 in July 2021 at Sabbie Nere, with 3.33 particles/kg of dry sediment. The highest concentration of LMPs near the dune can be attributed to the accumulation of sediments and small debris, including MPs, transported by both wind and sea. Furthermore, while LMPs were consistently detected at all sampling points at the Sabbie Nere site during all periods, no LMPs were found at points 1, 2, and 3 during the second sampling and at point 1 during the third sampling at the Gelso site.

**Fig. 6:**
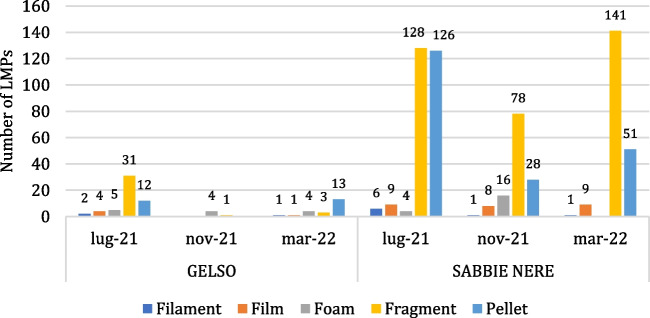
Classification of collected LMPs in the different categories for both sampling sites and periods

The categorization of collected LMPs (Fig. 6) showed that the detected categories were fragments (56%), followed by pellets (33%), foam (5%), films (5%), and filaments (2%).

Fragments are the only category detected in both sites and during all sampling periods, representing the most abundant one, except for the months of November 2021 and March 2022 at the Gelso site, where foam and pellets are the most numerous categories, respectively.

Regarding the possible sources of LMPs, the categories of fragments, foam, films, and filaments (which are of secondary origin) indicate that fragmentation has occurred from larger objects originating from the terrestrial environment through surface runoff, the fishing sector, or maritime navigation. Concerning fragments, due to their high heterogeneity, it is very difficult to make hypotheses about their emission source. Foams are often associated with fishing packaging, whereas filaments larger than 1 mm are usually linked to fishing gear such as nets or nylon threads. For pellets, on the contrary, being primary MPs, it is easier to trace back to their sources. The reasons they might be found in the environment are nearly threefold (Zhao et al. 2022): losses during pellet production, losses during transport, or losses during use for conversion into other objects. Given the discovery location, it is not possible to rule out any of these sources. However, since there are no plastic processing industries on the Aeolian Islands, the found pellets have undoubtedly traveled several miles, floating in the sea before washing ashore on Vulcano Island.

**Fig. 7:**
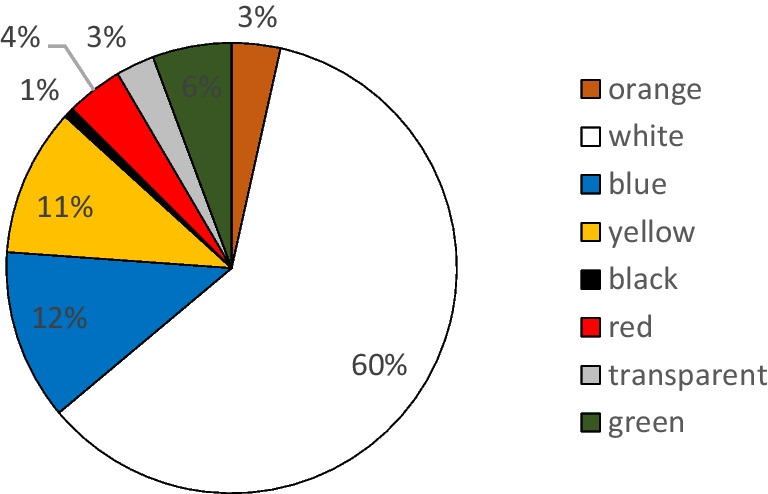
Color classification of LMPs (% in number)

Concerning the color classification of MPs (Fig. 7), white is the dominant color (60%), followed by blue (12%), yellow (11%), green (6%), red (4%), orange (3%), transparent (3%), and black (1%). No important variations in seasonality and sites are observed in terms of microplastic colors.

### Polymer identification

**Fig. 8:**
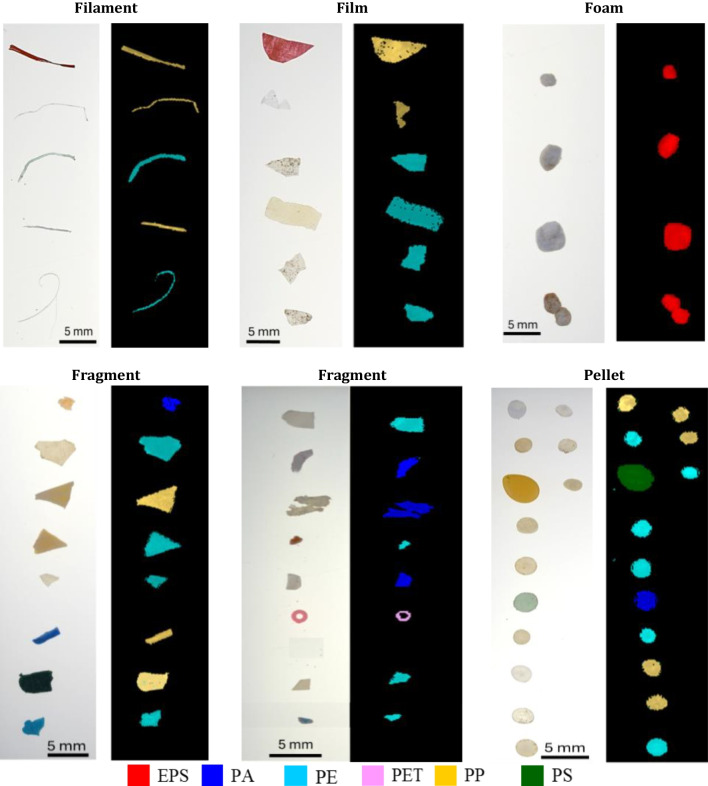
Representative source images and corresponding prediction maps obtained by HSI polymer classification for the different LMP categories (filament, film, foam, fragment, and pellet)

**Fig. 9:**
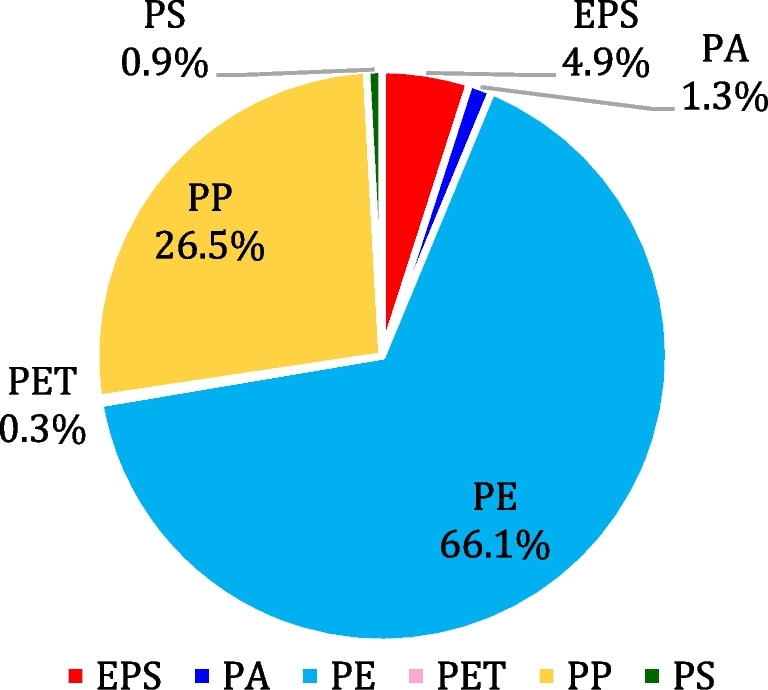
Overall distribution (% by number) of polymers constituting the collected LMPs identified by HSI

**Fig. 10:**
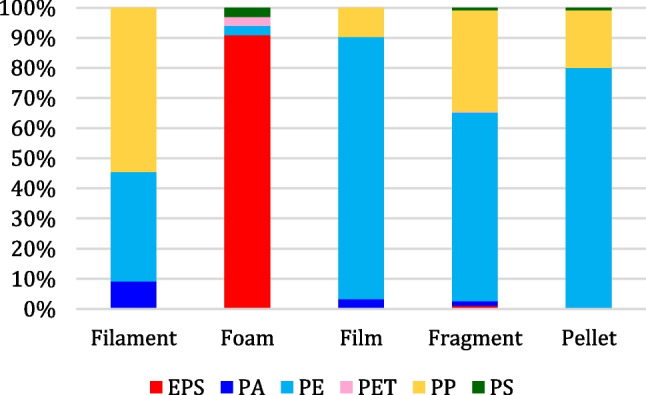
Distribution (% by number) of polymers detected in different microplastic categories

The results obtained by HSI for the classification of polymers constituting the LMP particles are shown in Fig. 8, in terms of representative prediction maps for the different MP categories and in Fig. 9 in terms of the overall distribution of the polymers, computed from all the prediction maps. Furthermore, the distribution of polymers among the different microplastic categories is shown in Fig. 10. From these results, it can be clearly stated that the most abundant polymer is PE, followed by PP, with smaller percentages of EPS, PA, PS, and PET.

Polymer distribution across the various categories revealed that PE and PP are the predominant polymers in filaments, films, fragments, and pellets (Fig. 10), while EPS is the most abundant in foams. Since PE and PP are the most produced polymers by industry for single-use products and packaging, they are also the ones most frequently found in the environment in the form of fragments, films, and filaments or due to direct discharge in the case of pellets. Conversely, an explanation for the large amount of EPS is that, thanks to its insulating and buoyant properties, it is widely used in small fishing communities, such as the island of Vulcano.

Furthermore, no important variations with respect to polymer distribution in seasonality and sites are observed.

### Morphological and morphometrical characterization

Regarding the results of morphological and morphometrical analysis, a summary is provided in Table 1.

**Table 1:** Summary of the main shape and size parameters of the investigated LMP particles belonging to the different categories obtained by morphological and morphometrical analysis

		**Area (mm**^**2**^**)**	**Aspect ratio**	**Feret min (mm)**	**Feret max (mm)**	**Feret mean (mm)**	**Perimeter (mm)**	**Roundness**
**Filament**	**Min**	0.1	-	-	4.6	-	6.8	-
	**Max**	6.9	-	-	5.0	-	21.4	-
	**Mean**	3.4	-	-	4.8	-	9.0	-
	**St. Dev**	3.5	-	-	1.0	-	6.5	-
**Film**	**Min**	3.8	1.1	1.8	2.3	2.5	9.9	0.3
	**Max**	25.0	3.5	2.5	5.0	3.1	25.6	0.9
	**Mean**	17.0	1.7	2.1	4.2	2.7	12.1	0.6
	**St. Dev**	6.1	0.6	1.2	0.9	1.5	4.5	0.2
**Foam**	**Min**	3.2	1.0	1.6	2.3	2.1	6.6	0.3
	**Max**	23.6	3.6	3.6	5.0	4.1	27.2	1.0
	**Mean**	11.4	1.7	3.2	4.0	3.1	15.2	0.7
	**St. Dev**	6.4	0.7	1.1	1.0	1.2	4.9	0.2
**Fragment**	**Min**	1.5	1.0	0.6	1.7	1.5	4.9	0.1
	**Max**	25.0	12.1	4.2	5.0	4.5	25.8	1.0
	**Mean**	11.3	1.8	3.0	3.8	3.1	14.3	0.6
	**St. Dev**	7.1	1.0	1.2	0.8	1.4	5.3	0.2
**Pellet**	**Min**	1.7	1.0	1.7	2.3	2.0	6.0	0.4
	**Max**	24.9	2.5	3.9	5.0	4.6	18.4	1.0
	**Mean**	13.1	1.2	2.2	4.4	4.1	9.3	0.8
	**St. Dev**	4.1	0.2	0.7	0.7	0.7	2.6	0.1

The results for the fragment category highlight a large variability for most size and shape parameters, with an *Area* ranging from 1.5 to 25 mm^2^ and a *Perimeter* from 4.9 to 25.8 mm. Considering the *Maximum Feret Diameter*, the fragment category exhibits the widest size distribution, with particles in all size classes from 1 to 5 mm. Additionally, fragments are, as expected, less circular (*Aspect ratio* from 1.0 to 12.1 and *Roundness* from 0.1 to 1.0) compared to pellets, which have high roundness (*Aspect ratio* from 1.0 to 2.5 and *Roundness* from 0.4 to 1.0).

The findings regarding filaments align with their elongated shape, as indicated by their small *Areas* ranging from 0.1 to 6.9 mm^2^ and large *Perimeters* ranging from 6.8 to 21.4 mm.

Regarding the mean of the *Maximum Feret Diameter* for category distribution, filaments (11 particles) show the highest value of 4.8 mm, followed by pellets (230 particles) with an average value of 4.4 mm, film (31 particles) with 4.2 mm, foam (33 particles) with 4.0 mm, and finally, fragments (382 particles) with 3.8 mm.

Fragments, the most abundant MP category (i.e., 382 particles, that is 56% of the total), were detected in both sites and during all sampling periods. The distribution of fragments based on the *Maximum Feret Diameter* correlated with polymer type, as shown in Fig. 10, highlighted that the number of collected MPs increases with their size, for both PE and PP.

**Fig. 11:**
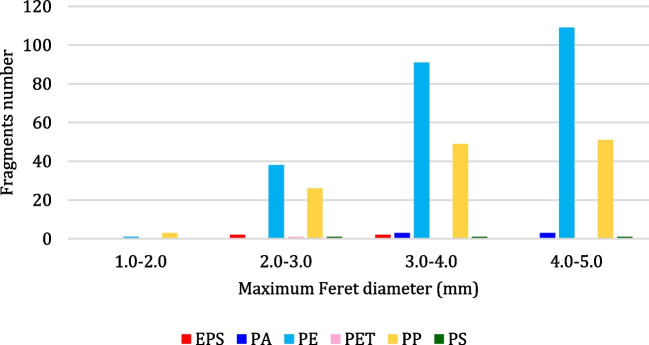
Number of fragments for each size range (in mm)

From Fig. 11, it can be noticed that the most abundant size class is 4–5 mm. PE and PP MPs are characterized by larger size variability than the other polymers. The *Maximum Feret Diameter* of PE (239 particles) ranges from 1.7 to 5 mm, while that of PP (129 particles) ranges from 1.9 to 5 mm. The *Maximum Feret Diameter* of the other polymers ranges as follows: for PA (6 particles) from 3.1 to 4.8 mm, for EPS (4 particles) from 2.7 to 4.0 mm, for PS (3 particles) from 2.4 to 4.1 mm, and for PET (1 particle) is 3.0 mm.

### MDII evaluation

The MDII calculated for the total sampled LMPs, for individual sites, and for the sampling periods are reported in Tables 2 and 3, respectively. Based on the results obtained for the MDII, it is possible to observe that Gelso is the site with the highest value, characterized by the lowest amount of LMPs and lower tourism and anthropogenic activity. This suggests that the MPs likely come from many distant sources compared to the sampling point(Li et al. 2021). Conversely, at Sabbie Nere, a more touristic site with a higher presence of LMPs, the MDII has the lowest value. This could mean that the sampled MPs come from a few sources very close to the sampling point. This seems to be in agreement with the previously hypothesized scenario: the Sabbie Nere site is more influenced, compared to Gelso, by local sources of MPs such as bathing activities and the presence of the little town.

**Table 2:** MDII of LMPs sampled in the sediment of beaches from Vulcano Island

Site	Total collected MPs (number)	MDII
Sabbie Nere	606	0.530019
Gelso	81	0.656282
**Total**	**687**	**0.554205**

**Table 3:** MDII of MPs sampled in sediments from beaches of Vulcano Island computed for the three different sampling periods

Period	Total collected MPs (number)	MDII
July 2021	327	0.562751
November 2021	136	0.531386
March 2022	224	0.520106

As shown in Table 3, the index has also been calculated for different sampling periods.

During the summer season, the highest index was observed, indicating greater heterogeneity among MPs, suggesting they have spent more time in the environment compared to those sampled in November 2021 and March 2022, which instead exhibit a slightly lower MDII. However, with no substantial variations among the three indices, drawing conclusions becomes challenging. Compared to other scientific studies, considering the highest MDII among the three seasons, the pollution level still appears to be moderately low.

Intensive tourist activities significantly contribute to plastic pollution (Abelouah et al. 2021) as tourist-generated waste on the coasts undergoes physical, chemical, and biological degradation, contributing to MP production. This is exactly what happens at Sabbie Nere, where the high influx of tourists and the morphology of the bay make it a greater accumulator of LMPs than Gelso. On the other hand, Gelso, with less tourism and a smaller bay that experiences almost complete sand turnover every year, has a reduced presence of LMPs.

### Comparison with other studies on microplastics collected on European coastal sands

The concentrations of MPs measured in this study carried out at Vulcano Island were compared with those found on the coasts of European beaches (Table 4). In all the compared case studies, the concentrations of MPs were higher than those observed in this study (0.92 particles/kg_dw). It is important to consider that the comparison of measured concentrations can be influenced by different sampling and analytical strategies. However, from our extensive work, carried out over multiple sampling periods, it is crucial to highlight the presence of few MPs in an area designated as a UNESCO heritage site, characterized by high biodiversity.

**Table 4:** Abundance of MPs in European sediment beach

Location	Particle size	Concentration (particles/kg_dw)	Prevalent polymer	Prevalent category	Prevalent color	References
Vulcano Island, Italy	1 mm – 5 mm	0.92 (0.27–1.35)	PE, PP	Fragments, pellets	White, blue, yellow	This study
Piombino, Italy	1 mm – 5 mm	1.98	Nylon, HDPE	Filaments, fragments	NR	(Mistri et al. 2021)
Portoferraio, Italy	1 mm – 5 mm	2.21	TPU, Nylon	Filaments, fragments	NR	(Mistri et al. 2021)
Talamone, Italy	1 mm – 5 mm	62	NR	Filaments, films	White, blue	(Cannas et al. 2017)
Groenendijk, Zoute, Belgium	38 μm – 1 mm	92.8	PP, PS	Fibres, granules	NR	(Claessens et al. 2011)
Bulk, Kiel fjord, Germany	0.2 – 5 mm	1.8	PS, PE	Fibres, fragments	Red, Blue	(Schröder et al. 2021)
Norderney, Germany	100 μm – 1 mm	1.45	PP, PE	Fibres	NR	(Dekiff et al. 2014)
Valletta, Malta	0.5 mm – 5 mm	35	NR	Fragments, filaments	NR	(Romeo et al. 2015)
Kaliningrad, Russia	1.3 – 5 mm	1.3–36.3	PS, PP	Foam, fragments	NR	(Esiukova, 2017)
Magrovica, Slovenia	63 μm - 5 mm	32.3	NR	Filaments, films	White, black	(Blašković et al. 2017)
Koper, Izola and Portoroz, Slovenia	0.25 mm – 5 mm	133.3	NR	Fibres, fragments	NR	(Laglbauer et al. 2014)

*Dw* dry weight, *HDPE* high-density polyethylene, *NR* not reported, *PE* polyethylene, *PP* polypropylene, *PS* polystyrene, *TPU* thermop polyurethane

In detail, as shown in Table 4, the highest concentration of MPs among the analyzed studies was 133 particles/kg_dw in Slovenia (Laglbauer et al. 2014). Elevated concentrations in the Mediterranean Sea were also found in Malta with 35 particles/kg_dw (Romeo et al. 2015). In Northern Europe, concentrations were detected in the Baltic Sea and the North Sea. In the Baltic Sea, concentrations ranged from 1.3 to 36.3 particles/kg_dw (Esiukova 2017; Schröder et al. 2021), while in the North Sea, concentrations reached 92.8 particles/kg_dw (Claessens et al. 2011).

Regarding Italy, the highest concentrations recorded among the investigated studies were found in the Tyrrhenian Sea, in Talamone (GR, Italy), with 62 particles/kg_dw.

Concerning the type of MPs, the predominant categories in the considered studies were fragments, followed by filaments/fibers, whereas in this work after fragments, the most abundant category was the pellet. The reason why pellets are in second place in this study can be explained by considering the almost total absence of fibers/filaments. Such category, mainly coming from urban and fishing sectors, is present in limited quantities due to the small local population. This contrasts with the considered European areas, where it is more common to find a greater quantity of fibers/filaments on sandy shores. The most diffused polymers were PE and PP, while white and blue were the most common colors, both results agreeing with the findings of this work. White is the predominant color due not only to a higher production of white items that end up in the sea but also to a yellowish-white discoloration of all colored plastics (Khoironi et al. 2020; Pfohl et al. 2022).

## Conclusions

In this work, the monitoring and characterization of LMPs collected from the sandy beaches of Vulcano Island (Aeolian Islands, Sicily, Italy) were carried out for the first time.

The application of HSI as a rapid and non-destructive method for classifying the MPs found in marine sediments for environmental monitoring represents an innovative approach in this field. This approach not only demonstrated high efficiency and accuracy in identifying polymer types but also proved to be a valuable tool for large-scale environmental monitoring, allowing for more comprehensive and timely assessments of microplastic pollution.

The data obtained from the three sampling periods in terms of concentration provide a robust estimate of microplastic abundance on Vulcano Island. The average abundance of sampled LMPs was 0.92 particles/kg of dry sediment, and the concentration was higher on the more touristy Sabbie Nere (1.35 particles/kg of dry sediment) compared to the less touristy Gelso (0.27 particles/kg of dry sediment).

Furthermore, a higher MDII at the Gelso site suggests the presence of numerous contamination sources, attributed to a greater polymer heterogeneity, category, and color compared to Sabbie Nere, which instead exhibits a lower MDII, indicating a lower presence of contamination sources. Since Sabbie Nere is the most touristic beach on the island and is located closer to the inhabited center, it exhibits less variability as tourism predominates as the main and sole source of contamination. The robustness of the data for Gelso is lower due to the variability of the beach and the instability of microplastics (MP) from year to year. The dynamic process of deposition and erosion stabilizes MP levels at lower values compared to Sabbie Nere. The rapid variability and high renewal rate of MPs in Gelso prevent the selection of less mobile plastics, a phenomenon possible in more stable sites like Sabbie Nere. To attribute the results to this phenomenon, monitoring should be extended over several years.

The results of this study confirm the data found in the literature regarding microplastic contamination of beaches, namely fragments and pellets are among the most common categories found in sand, and the most abundant polymers are PE and PP. In all analyzed categories, the most abundant polymers were PE (66.1%) and PP (26.5%), except for foam, which was identified as EPS. These findings highlight the pervasive nature of certain types of polymers in marine environments and underscore the need for targeted pollution management strategies focusing on these contaminants.

Further studies will be carried out to detect and classify the SMPs directly in the sand background by HSI.

## Data Availability

Datasets generated during the current study are available from the corresponding author on reasonable request.
